# Supplementary material for: Periphery-Fused Chiral A2B-Type Subporphyrin
Source: Molecules. 2021 Feb 20;26(4):1140. doi: 10.3390/molecules26041140 (PMC7924371; doi:10.3390/molecules26041140)
Supplement: Supplementary file 1 [file molecules-26-01140-s001.pdf]

Supplemental Material

for

Periphery-Fused Chiral A_2B-Type Subporphyrin

Shoma Hirokawa [1], Nagao Kobayashi [1,2,*] and Soji Shimizu [3,*]

[1]Department of Chemistry, Graduate School of Science, Tohoku University, Sendai 980-8578, Japan

[2]Faculty of Textile Science and Technology, Shinshu University, Ueda 386-8567, Japan

[3]Department of Chemistry and Biochemistry, Graduate School of Engineering and Center for Molecular Systems (CMS), Kyushu University, Fukuoka 819-0395, Japan

*Correspondence: nagaok@shinshu-u.ac.jp (N.K.); ssoji@cstf.kyushu-u.ac.jp (S.S.) Tel.: +81-92-802-2866 (S.S.)

Contents:

i. HR-MALDI-FT-ICR-MS Spectra

ii. ^{1}H NMR Spectra

iii. Racemization of **5** at 40 °C

iv. Theoretical Calculations

v. Analyses of Transition Dipole Moments

vi. Theoretical ^{1}H NMR Chemical Shifts of **6**'

vii. Appendix

i. HR-MALDI-FT-ICR-MS Spectra

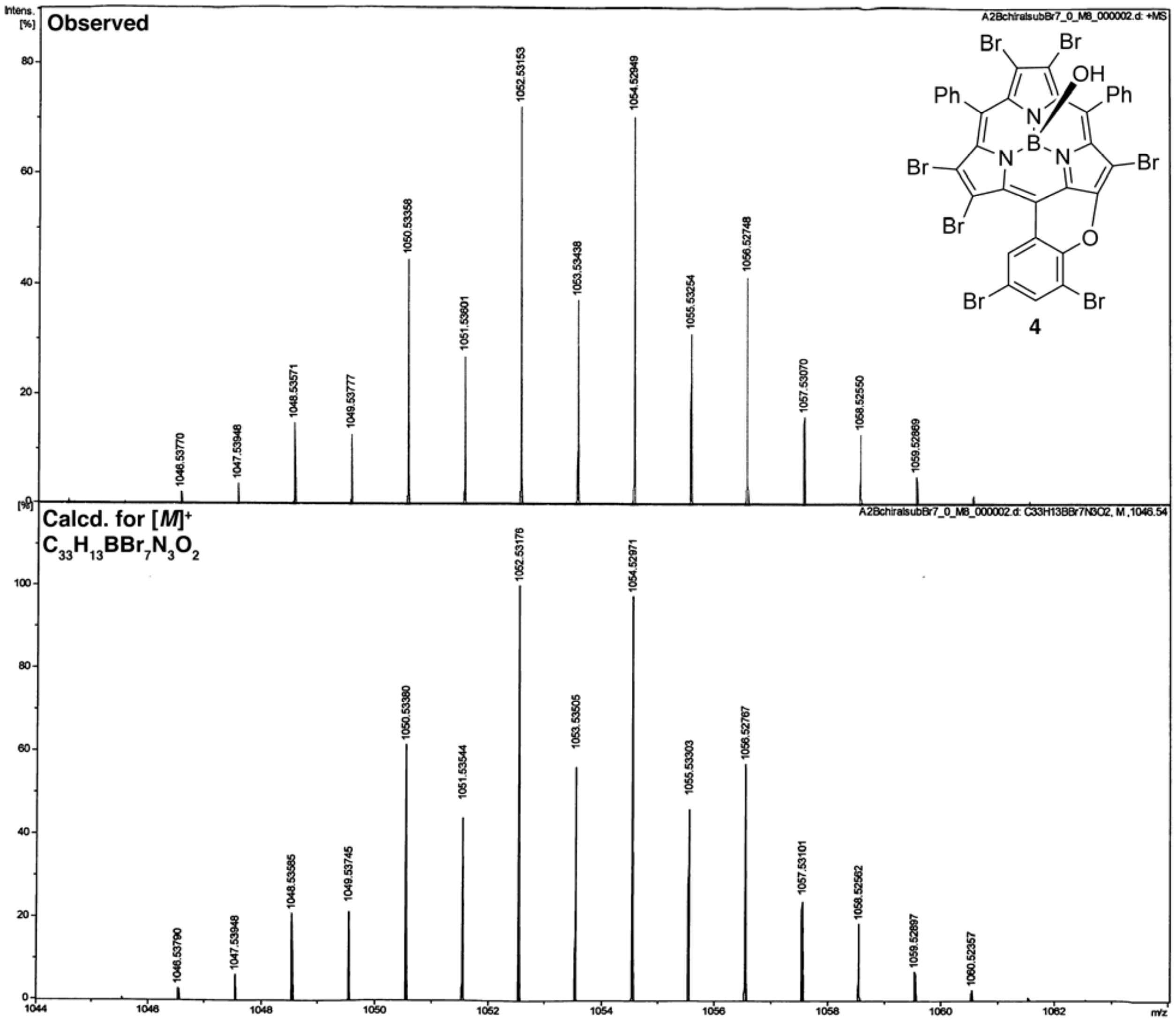

Figure S1. High-resolution mass spectrum of **4**, observed spectrum (top) and isotopic patterns calculated for $C_{33}H_{13}BBr_7N_3O_2$ as a $[M]^+$ ion (bottom).

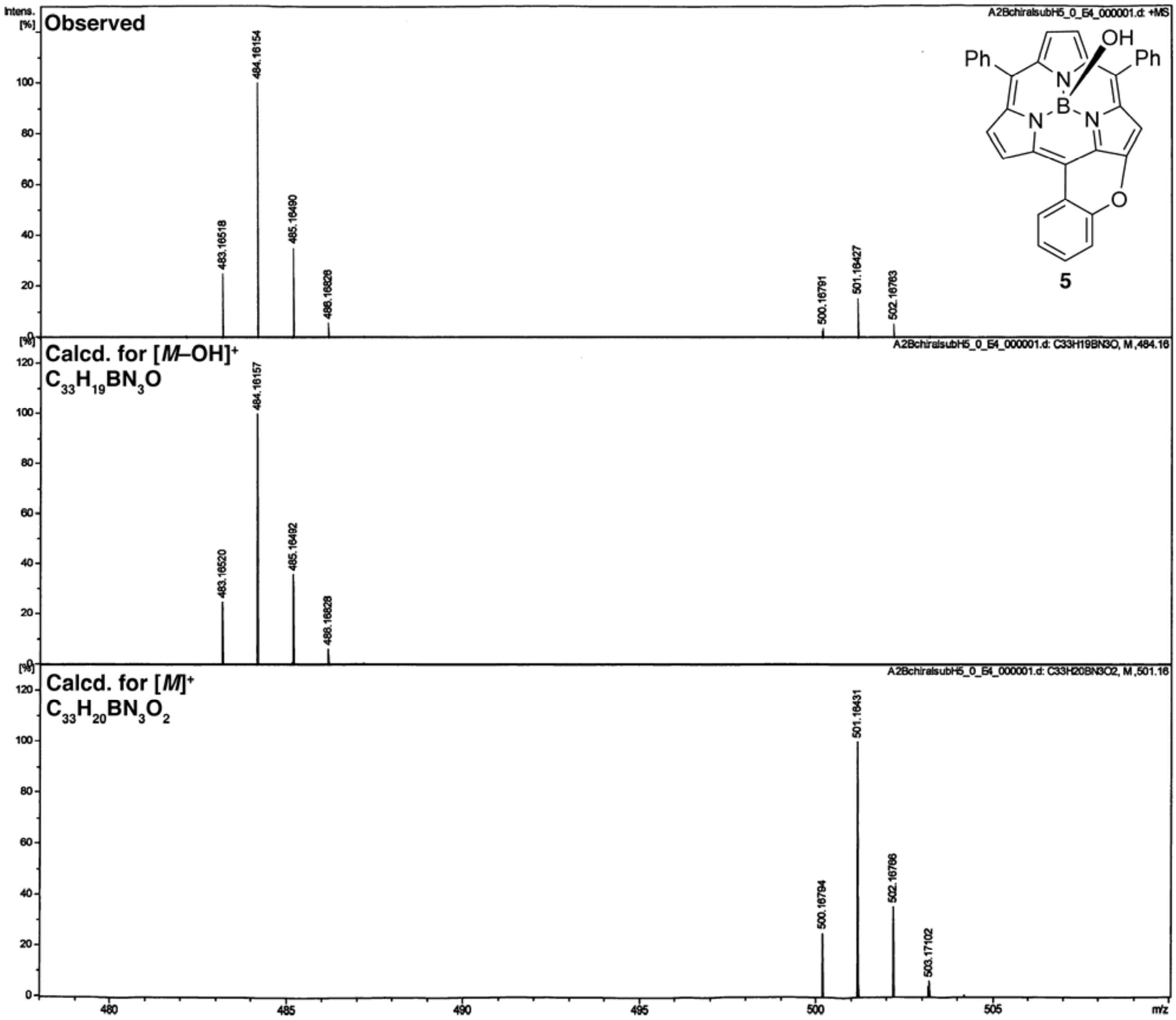

Figure S2. High-resolution mass spectrum of **5**, observed spectrum (top) and isotopic patterns calculated for $C_{33}H_{19}BN_3O$ as a [*M*–OH]$^+$ ion (middle) and $C_{33}H_{20}BN_3O_2$ as a [*M*]$^+$ ion (bottom).

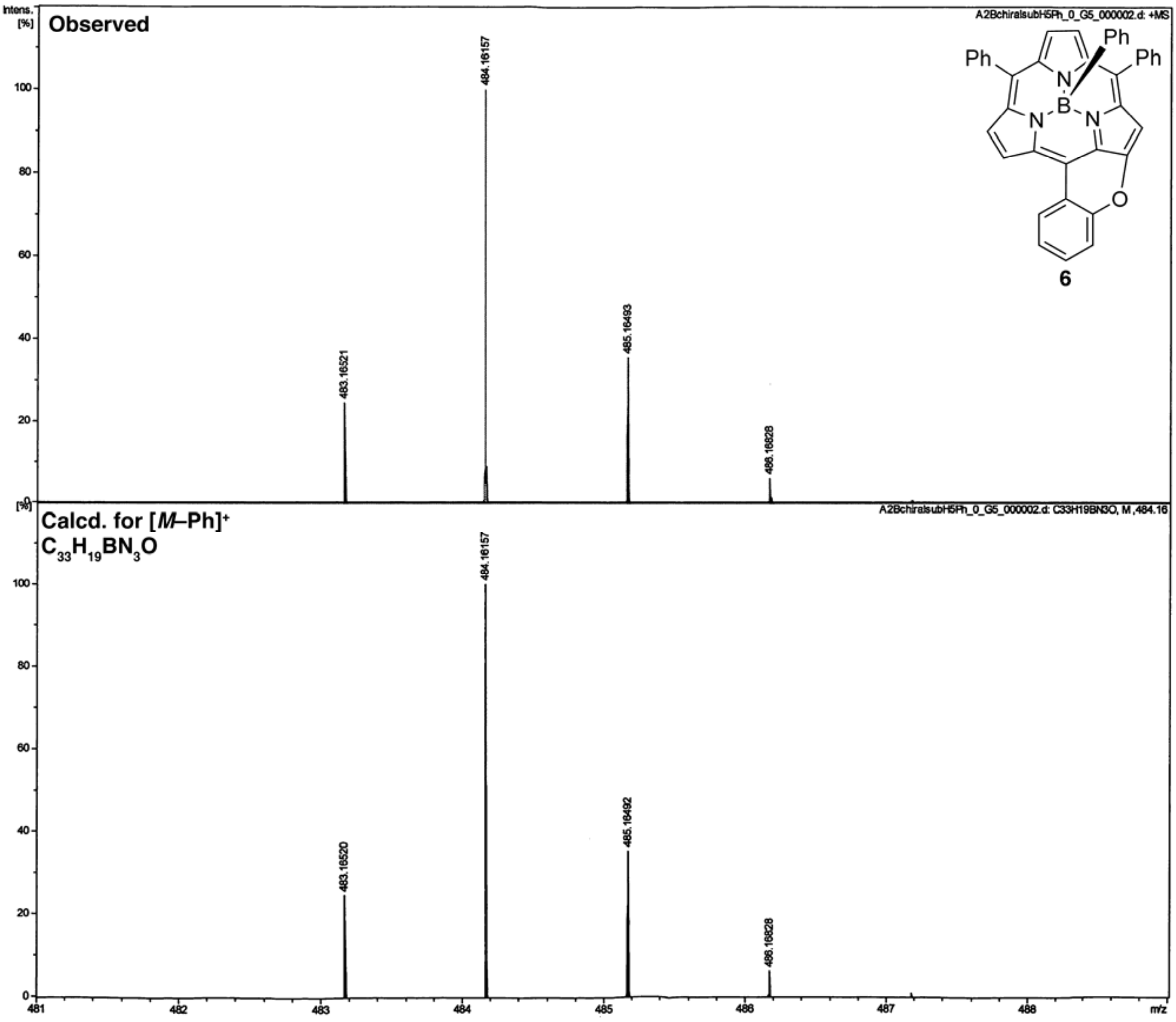

Figure S3. High-resolution mass spectrum of **6**, observed spectrum (top) and isotopic patterns calculated for $C_{33}H_{19}BN_3O$ as a [*M*–Ph]+ ion (bottom).

ii. ¹H NMR Spectra

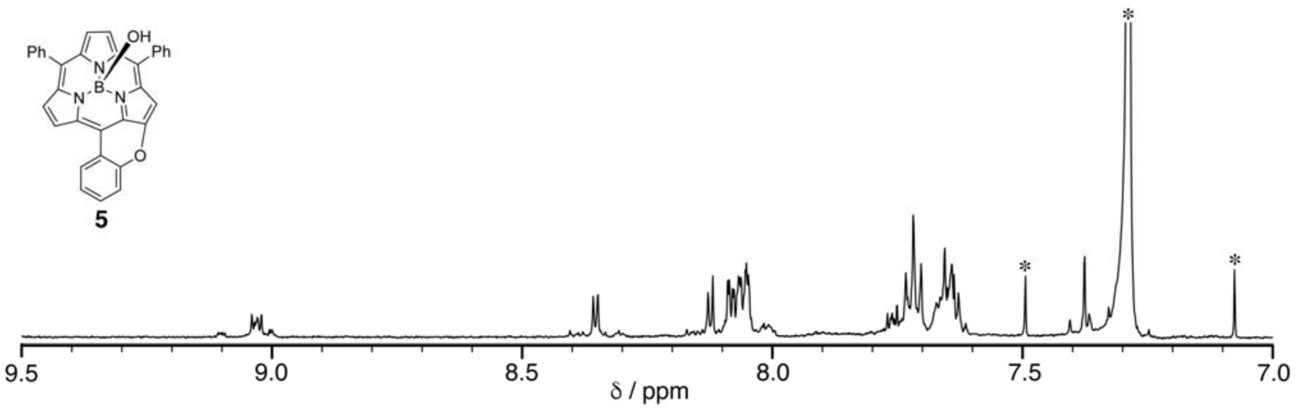

Figure S4. ¹H NMR spectrum of **5** in $CDCl_3$. ∗ indicates residual solvent signals.

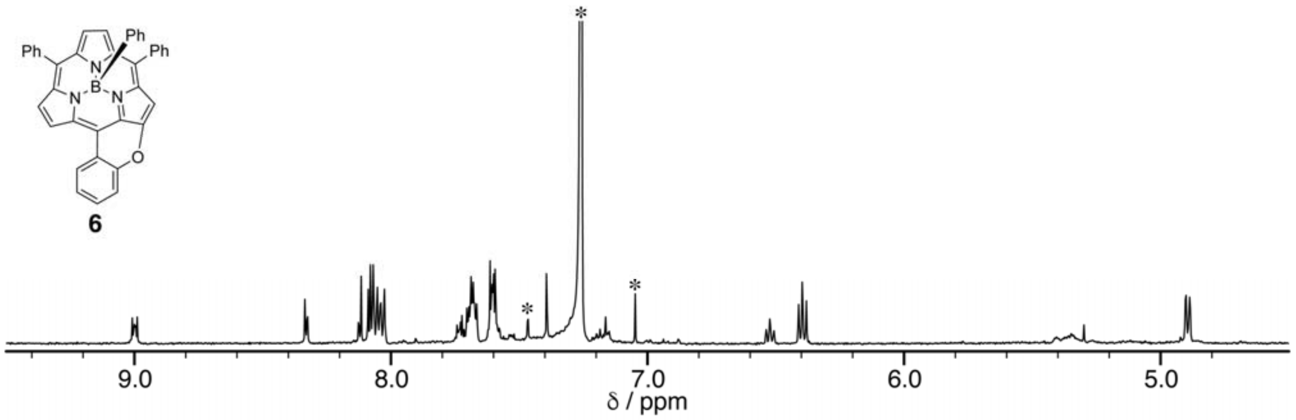

Figure S5. ¹H NMR spectrum of **6** in $CDCl_3$. ∗ indicates residual solvent signals.

iii. Racemization of 5 at 40 °C

After chiral resolution of **5**, the CD spectrum was measured. Because of the incomplete separation and racemization during removing solvent from the eluted fraction, the CD intensities were relatively low compared with those of **4** and **6**. By heating the sample solution at 40 °C, the CD intensities gradually decreased due to racemization in solution.

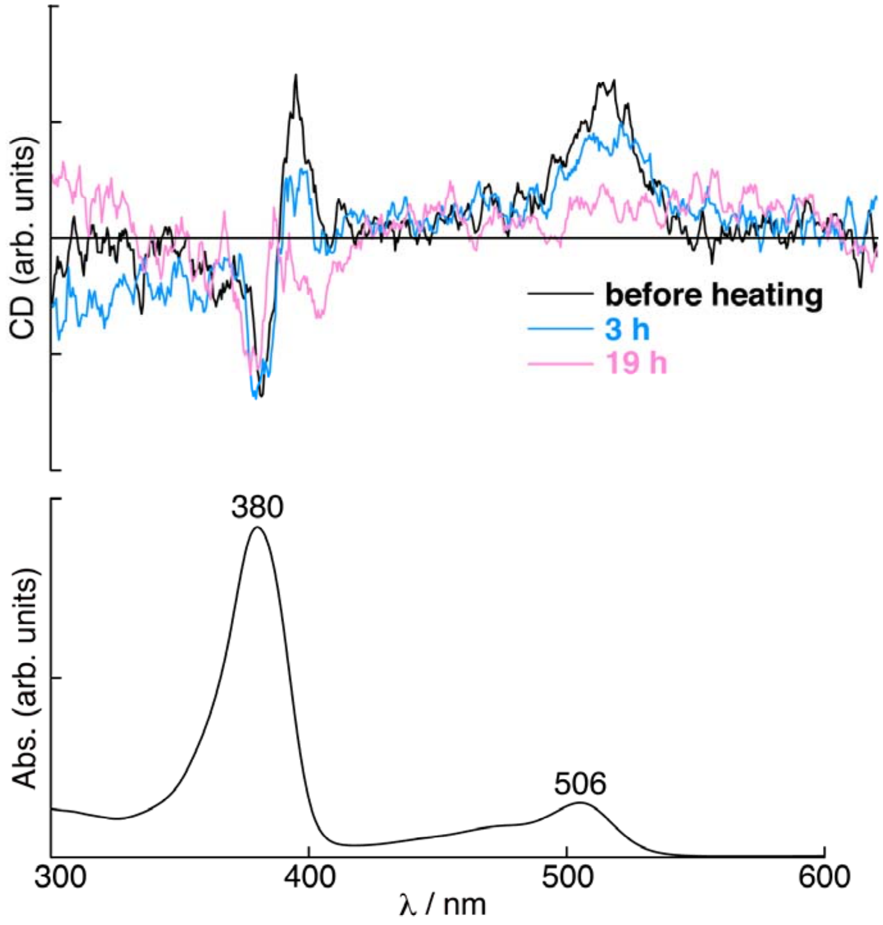

Figure S6. CD (top) and UV/vis absorption (bottom) spectra of **5** in CH_2Cl_2 after chiral resolution (black line) and after heating at 40 °C for 3 h (aqua blue line) and 19 h (pink line).

iv. Theoretical Calculations

Table S1. Selected transition wavelengths, oscillator strengths, rotatory strengths, and major contributions of **4'** calculated by the TDDFT method at the B3LYP/6-311G(d,p) level.

No.	wavelength [nm]	f[a]	R (velocity)[b]	Major contributions[d]
1	478	0.14	2.0 (**4'*P***)[c]	H–1→L+1 (19%), HOMO→LUMO (77%)
			–2.0 (**4'*M***)[c]	
2	463	0.02	–0.3 (**4'*P***)	H–1→LUMO (38%), HOMO→L+1 (59%)
			0.3 (**4'*M***)	
3	401	0.05	–9.6 (**4'*P***)	H–2→LUMO (67%), H–2→L+1 (12%), H–1→L+1 (12%)
			9.6 (**4'*M***)	
4	389	0.13	6.3 (**4'*P***)	H–3→LUMO (34%), H–2→LUMO (11%), H–2→L+1 (19%), H–1→LUMO (20%)
			–6.3 (**4'*M***)	
5	384	0.07	36.7 (**4'*P***)	H–3→LUMO (56%), H–2→L+1 (19%)
			–36.7 (**4'*M***)	
6	373	0.01	–1.1 (**4'*P***)	H–6→LUMO (12%), H–4→LUMO (64%)
			1.1 (**4'*M***)	
7	365	0.11	–26.1 (**4'*P***)	H–5→LUMO (50%), H–3→L+1 (25%)
			26.1 (**4'*M***)	
8	361	0.53	57.2 (**4'*P***)	H–3→L+1 (25%), H–1→L+1 (36%), HOMO→LUMO (12%)
			–57.2 (**4'*M***)	
9	357	0.16	–21.7 (**4'*P***)	H–5→LUMO (12%), H–4→L+1 (13%), H–3→L+1 (33%), H–2→L+1 (12%)
			21.7 (**4'*M***)	
10	354	0.52	–22.3 (**4'*P***)	H–5→LUMO (22%), H–3→L+1 (10%), H–2→L+1 (20%), H–1→LUMO (13%)
			22.3 (**4'*M***)	
11	351	0.08	–30.5 (**4'*P***)	H–6→LUMO (66%)
			30.5 (**4'*M***)	
12	347	0.12	–23.0 (**4'*P***)	H–6→L+1 (14%), H–4→LUMO (13%), H–4→L+1 (46%)
			23.0 (**4'*M***)	

[a] Oscillator strength. [b] Rotatory strength [10^{-40} erg•esu•cm•G^{-1}]. [c] **4'*P*** and **4'*M*** denote ***P*** and ***M*** isomers, respectively.
[d] H and L denote the HOMO and LUMO, respectively.

Table S2. Selected transition wavelengths, oscillator strengths, rotatory strengths, and major contributions of **6'** calculated by the TDDFT method at the B3LYP/6-311G(d,p) level.

No.	wavelength [nm]	f[a]	R (velocity)[b]	Major contributions[d]
1	477	0.16	7.57 (**6'*P***)[c]	H–1→L+1 (18%), HOMO→LUMO (80%)
			–7.57 (**6'*M***)[c]	
2	461	0.09	5.36 (**6'*P***)	H–1→LUMO (28%), HOMO→L+1 (70%)
			–5.36 (**6'*M***)	
3	365	0.61	25.9 (**6'*P***)	H–2→L+1 (12%), H–1→LUMO (52%), HOMO→L+1 (22%)
			–25.9 (**6'*M***)	
4	361	0.43	–14.3 (**6'*P***)	H–2→LUMO (14%), H–1→L+1 (56%), HOMO→LUMO (12%)
			14.3 (**6'*M***)	
5	342	0.21	–13.2 (**6'*P***)	H–2→LUMO (55%), H–2→L+1 (23%), H–1→L+1 (13%)
			13.2 (**6'*M***)	
6	335	0.16	–36.0 (**6'*P***)	H–3→LUMO (13%), H–2→LUMO (21%), H–2→L+1 (51%)
			36.0 (**6'*M***)	

[a] Oscillator strength. [b] Rotatory strength [10^{-40} erg•esu•cm•G^{-1}]. [c] **6'*P*** and **6'*M*** denote ***P*** and ***M*** isomers, respectively.
[d] H and L denote the HOMO and LUMO, respectively.

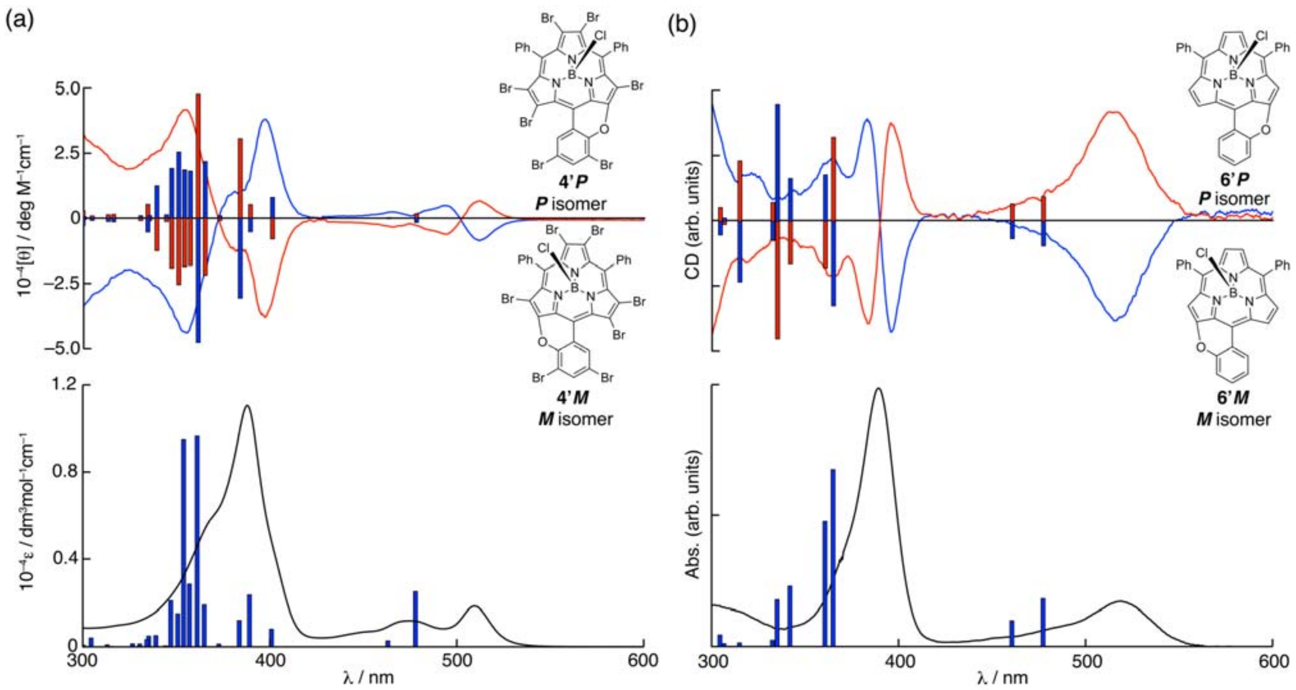

Figure S7. Theoretical absorption and CD spectra of the ***P*** (red bars) and ***M*** (blue bars) isomers of (a) **4'** and (b) **6'** calculated by the TDDFT method at the B3LYP/6-311G(d,p) level. The observed absorption and CD spectra of **4** and **6** are overlapping (**4Fr1** and **6Fr1**: blue line and **4Fr2** and **6Fr2**: red line).

v. Analyses of Transition Dipole Moments

Transition electric (TEDM) and transition magnetic (TMDM) dipole moments were generated from the TDDFT data by Multiwfn (Lu T.; Chen F., Multiwfn: A multifunctional wavefunction analyzer. *J. Comput. Chem.* **2012**, *33*, 580–592.) and visualized by VMD (Humphrey, W.; Dalke, A.; Schulten, K., VMD - Visual Molecular Dynamic. *J. Molec. Graphics* **1996**, *14*, 33–38.).

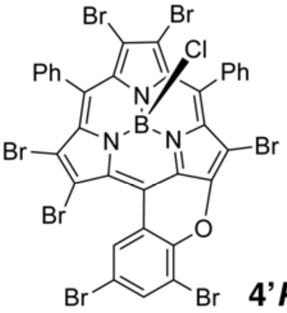

4'*P*

1st transition (λ = 478 nm)

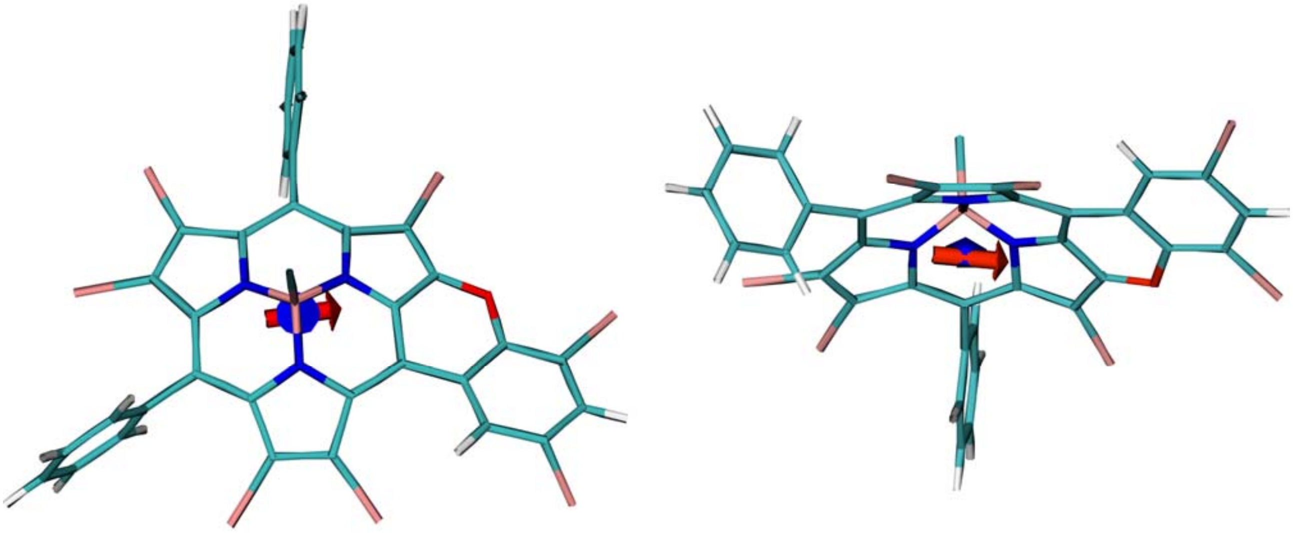

2nd transition (λ = 463 nm)

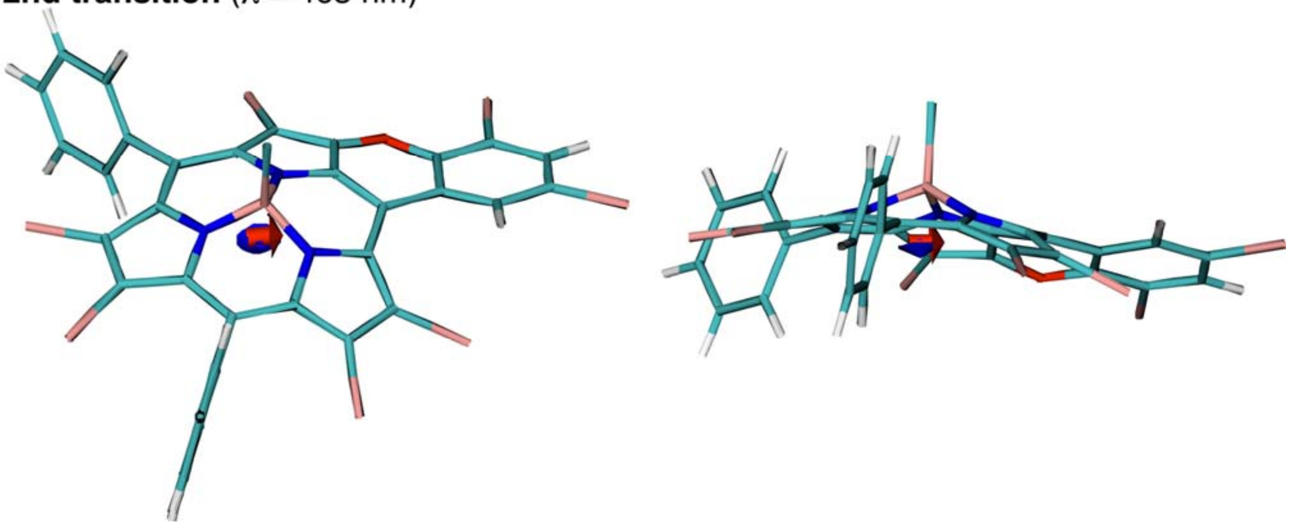

Figure S8. TEDM (red arrow) and TMDM (blue arrow) of **4'*P***, top view: left and side view: right.

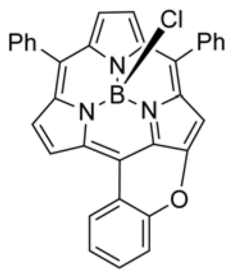

6'*P*

1st transition (λ = 477 nm)

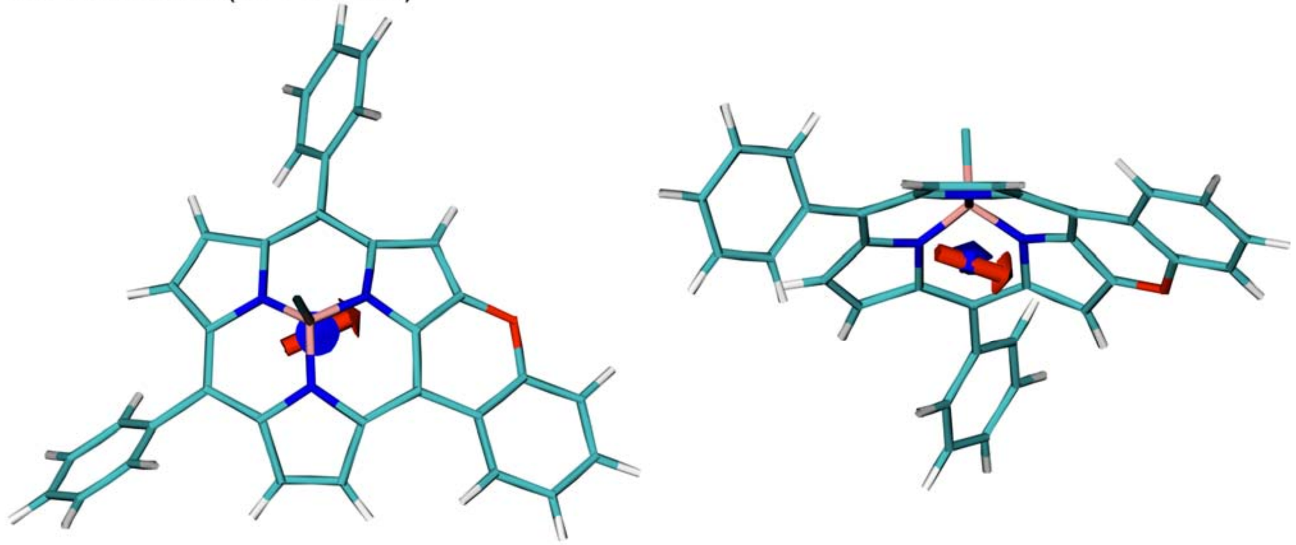

2nd transition (λ = 461 nm)

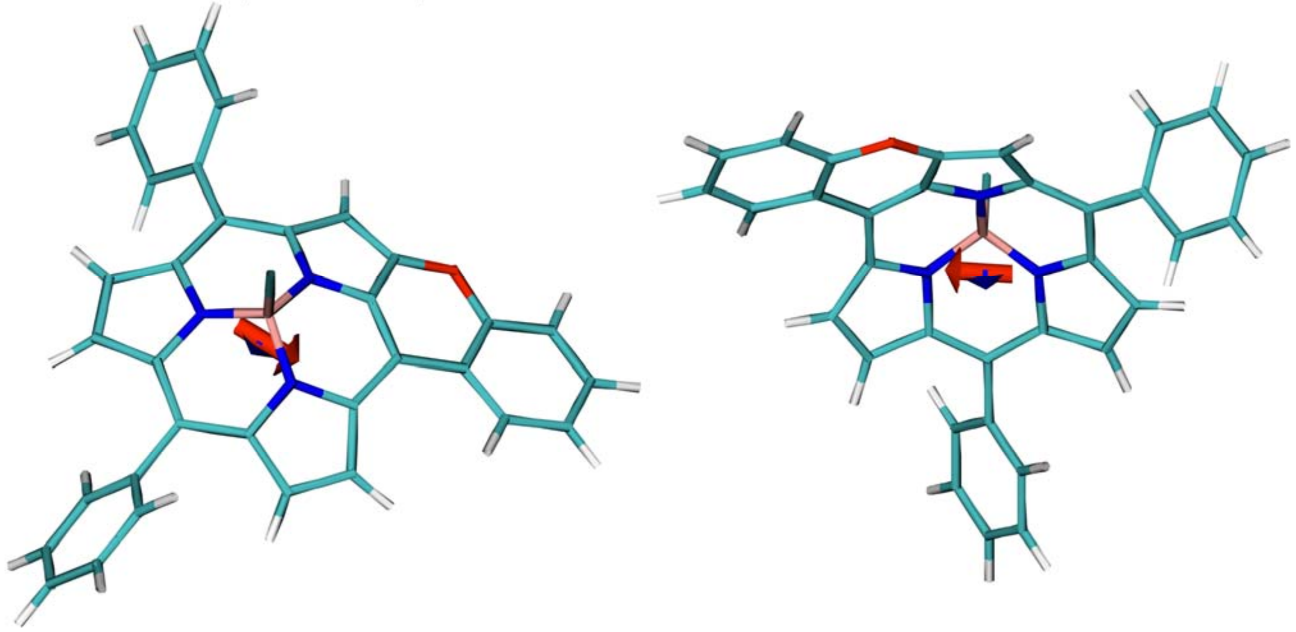

Figure S9. TEDM (red arrow) and TMDM (blue arrow) of **6'*P***, top view: left and side view: right.

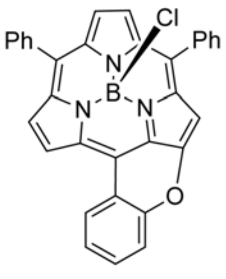

6'*P*-90

1st transition (λ = 453 nm)

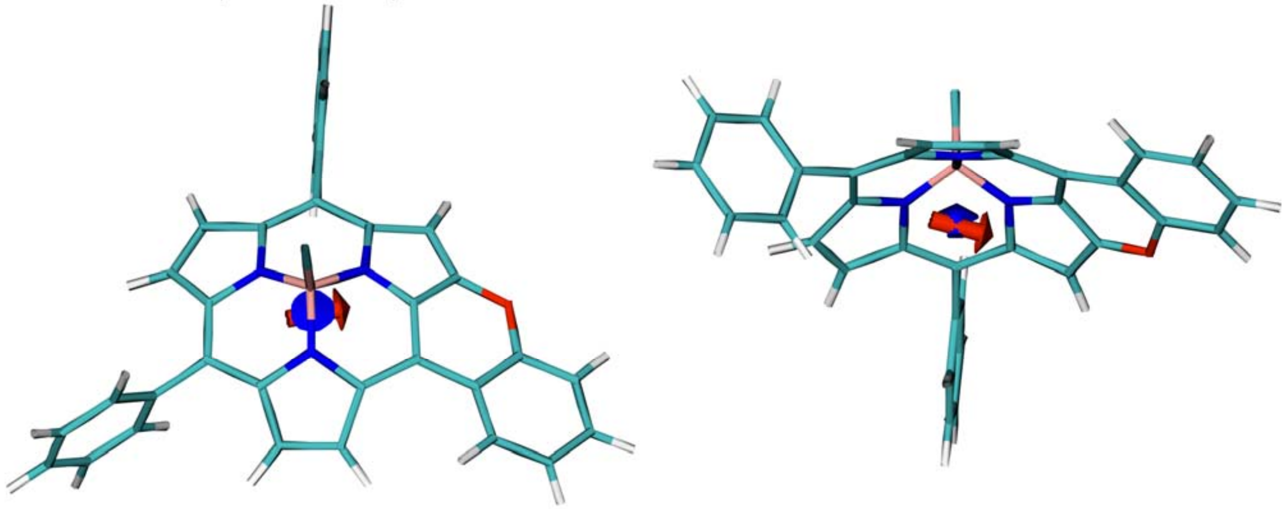

2nd transition (λ = 442 nm)

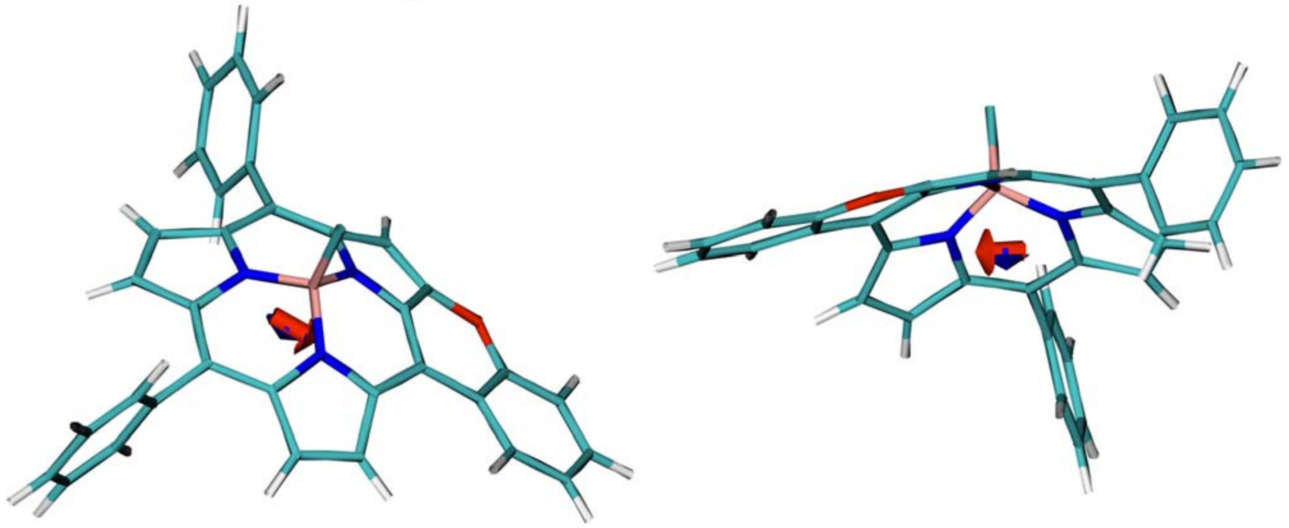

Figure S10. TEDM (red arrow) and TMDM (blue arrow) of **6'*P*-90**, top view: left and side view: right.

vi. Theoretical ¹H NMR Chemical Shifts of 6'

Theoretical ¹H NMR chemical shifts of **6'** were calculated at the B3LYP/6-311G(d,p) level with gauge-including atomic orbitals (GIAOs) using the DFT-optimized geometries.

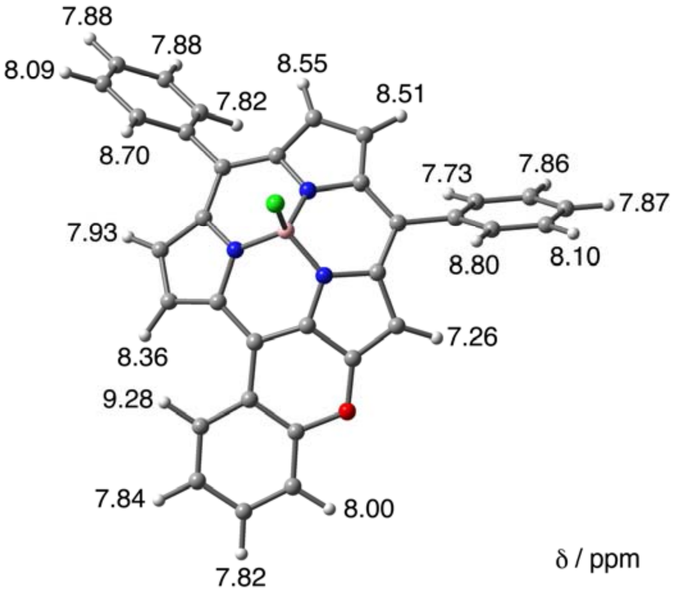

Figure S11. Theoretical ¹H NMR chemical shifts of **6'**.

vii. Appendix

Table Appx-1. Cartesian coordinates of the DFT optimized geometry of **4'*P*** at the B3LYP/6-311G(d,p).

	Symbol	X	Y	Z
1	O	3.130168	2.330189	−0.249013
2	N	0.049665	1.125317	0.845061
3	N	−0.162673	−1.248635	0.71757
4	N	−2.090338	0.178962	0.693505
5	C	−0.401633	2.374013	0.491827
6	C	0.788207	3.102872	0.081293
7	C	1.847078	2.209075	0.141335
8	C	1.351001	0.961429	0.580153
9	C	2.012651	−0.265577	0.468369
10	C	1.174004	−1.429757	0.4225
11	C	1.247131	−2.768726	−0.095074
12	C	−0.033782	−3.309412	−0.11288
13	C	−0.953276	−2.30806	0.355175
14	C	−2.37546	−2.165289	0.305634
15	C	−2.926959	−0.867723	0.419139
16	C	−4.202813	−0.243354	0.140322
17	C	−4.027622	1.131206	0.164884
18	C	−2.638625	1.413076	0.464313
19	C	−1.805383	2.565955	0.401765
20	C	3.457924	−0.067029	0.284927
21	C	3.938035	1.226568	−0.073969
22	C	5.307427	1.437803	−0.263878
23	C	6.224023	0.416674	−0.059511
24	H	7.279365	0.597759	−0.203571
25	C	5.757367	−0.825484	0.361474
26	C	4.407414	−1.068669	0.53833
27	H	4.077091	−2.030845	0.894189
28	C	−3.243075	−3.346589	0.020888
29	C	−3.629061	−4.189856	1.066978
30	H	−3.298073	−3.974605	2.076433
31	C	−4.434837	−5.297096	0.815539
32	H	−4.730603	−5.94324	1.634156
33	C	−4.857646	−5.573837	−0.482649
34	H	−5.482969	−6.437669	−0.678014
35	C	−4.475085	−4.737198	−1.52936
36	H	−4.8006	−4.948052	−2.541699
37	C	−3.674033	−3.627947	−1.278914
38	H	−3.374976	−2.979452	−2.094302
39	C	−2.35649	3.923235	0.113197
40	C	−2.559997	4.827546	1.159849
41	H	−2.33148	4.528127	2.176237
42	C	−3.057246	6.102003	0.900892
43	H	−3.214787	6.794033	1.72028
44	C	−3.348318	6.486662	−0.40596
45	H	−3.732895	7.480133	−0.607209
46	C	−3.141546	5.592001	−1.45406
47	H	−3.362779	5.88719	−2.473445
48	C	−2.649475	4.316561	−1.196131
49	H	−2.487257	3.622303	−2.01259
50	B	−0.763533	−0.013832	1.317595
51	Cl	−0.848349	−0.115798	3.218485
52	Br	0.933371	4.870159	−0.543065
53	Br	−5.392196	2.384448	−0.146188
54	Br	−5.834776	−1.103719	−0.210699
55	Br	−0.445451	−5.031459	−0.743597
56	Br	2.742039	−3.642743	−0.838681
57	Br	5.942952	3.155685	−0.788816
58	Br	7.018696	−2.219784	0.714705

Table Appx-2. Cartesian coordinates of the DFT optimized geometry of **4'*M*** at the B3LYP/6-311G(d,p).

	Symbol	X	Y	Z
1	O	−3.130168	2.330189	−0.249013
2	N	−0.049665	1.125317	0.845061
3	N	0.162673	−1.248635	0.71757
4	N	2.090338	0.178962	0.693505
5	C	0.401633	2.374013	0.491827
6	C	−0.788207	3.102872	0.081293
7	C	−1.847078	2.209075	0.141335
8	C	−1.351001	0.961429	0.580153
9	C	−2.012651	−0.265577	0.468369
10	C	−1.174004	−1.429757	0.4225
11	C	−1.247131	−2.768726	−0.095074
12	C	0.033782	−3.309412	−0.11288
13	C	0.953276	−2.30806	0.355175
14	C	2.37546	−2.165289	0.305634
15	C	2.926959	−0.867723	0.419139
16	C	4.202813	−0.243354	0.140322
17	C	4.027622	1.131206	0.164884
18	C	2.638625	1.413076	0.464313
19	C	1.805383	2.565955	0.401765
20	C	−3.457924	−0.067029	0.284927
21	C	−3.938035	1.226568	−0.073969
22	C	−5.307427	1.437803	−0.263878
23	C	−6.224023	0.416674	−0.059511
24	H	−7.279365	0.597759	−0.203571
25	C	−5.757367	−0.825484	0.361474
26	C	−4.407414	−1.068669	0.53833
27	H	−4.077091	−2.030845	0.894189
28	C	3.243075	−3.346589	0.020888
29	C	3.629061	−4.189856	1.066978
30	H	3.298073	−3.974605	2.076433
31	C	4.434837	−5.297096	0.815539
32	H	4.730603	−5.94324	1.634156
33	C	4.857646	−5.573837	−0.482649
34	H	5.482969	−6.437669	−0.678014
35	C	4.475085	−4.737198	−1.52936
36	H	4.8006	−4.948052	−2.541699
37	C	3.674033	−3.627947	−1.278914
38	H	3.374976	−2.979452	−2.094302
39	C	2.35649	3.923235	0.113197
40	C	2.559997	4.827546	1.159849
41	H	2.33148	4.528127	2.176237
42	C	3.057246	6.102003	0.900892
43	H	3.214787	6.794033	1.72028
44	C	3.348318	6.486662	−0.40596
45	H	3.732895	7.480133	−0.607209
46	C	3.141546	5.592001	−1.45406
47	H	3.362779	5.88719	−2.473445
48	C	2.649475	4.316561	−1.196131
49	H	2.487257	3.622303	−2.01259
50	B	0.763533	−0.013832	1.317595
51	Cl	0.848349	−0.115798	3.218485
52	Br	−0.933371	4.870159	−0.543065
53	Br	5.392196	2.384448	−0.146188
54	Br	5.834776	−1.103719	−0.210699
55	Br	0.445451	−5.031459	−0.743597
56	Br	−2.742039	−3.642743	−0.838681
57	Br	−5.942952	3.155685	−0.788816
58	Br	−7.018696	−2.219784	0.714705

Table Appx-3. Cartesian coordinates of the DFT optimized geometry of **6'*P*** at the B3LYP/6-311G(d,p).

	Symbol	X	Y	Z
1	O	4.006231	−1.506737	−0.57379
2	N	1.175372	0.072354	0.736121
3	N	−0.811272	−1.268946	0.599435
4	N	−0.928854	1.118988	0.530282
5	C	1.83706	1.209223	0.34161
6	C	3.12829	0.786483	−0.146387
7	H	3.889674	1.425894	−0.56072
8	C	3.119284	−0.59858	−0.104433
9	C	1.868403	−1.031013	0.403963
10	C	1.319336	−2.303976	0.227308
11	C	−0.105252	−2.399061	0.249192
12	C	−1.067514	−3.300762	−0.306163
13	H	−0.855206	−4.274832	−0.716626
14	C	−2.292964	−2.660489	−0.333443
15	H	−3.207351	−3.042815	−0.758866
16	C	−2.116448	−1.341308	0.195769
17	C	−2.907619	−0.15548	0.089515
18	C	−2.254815	1.095697	0.19031
19	C	−2.572789	2.449761	−0.18687
20	H	−3.54459	2.805086	−0.489687
21	C	−1.409501	3.188263	−0.152788
22	H	−1.306346	4.226966	−0.422343
23	C	−0.331686	2.317273	0.248842
24	C	1.087335	2.412013	0.210326
25	C	2.319902	−3.284496	−0.191412
26	C	3.617515	−2.838359	−0.5613
27	C	4.600674	−3.73571	−0.967897
28	H	5.570756	−3.342137	−1.244787
29	C	4.326188	−5.095102	−1.009314
30	H	5.097162	−5.788295	−1.323914
31	C	3.06517	−5.564287	−0.634123
32	H	2.853284	−6.62668	−0.645966
33	C	2.085688	−4.670515	−0.230941
34	H	1.122273	−5.041103	0.094813
35	C	−4.338638	−0.268074	−0.27847
36	C	−5.193027	−1.078828	0.484636
37	H	−4.796713	−1.595543	1.350922
38	C	−6.540182	−1.200008	0.156815
39	H	−7.18592	−1.823482	0.764982
40	C	−7.059648	−0.517431	−0.941058
41	H	−8.109127	−0.611673	−1.195713
42	C	−6.220249	0.283752	−1.712646
43	H	−6.612639	0.806481	−2.577835
44	C	−4.872739	0.40632	−1.387124
45	H	−4.220653	1.004195	−2.012385
46	C	1.783152	3.673687	−0.136073
47	C	2.850172	4.120481	0.658651
48	H	3.132785	3.547319	1.534056
49	C	3.525047	5.29768	0.349466
50	H	4.340698	5.630076	0.981771
51	C	3.149864	6.050775	−0.761033
52	H	3.674956	6.968267	−1.001095
53	C	2.098389	5.613906	−1.564326
54	H	1.809893	6.185222	−2.439641
55	C	1.423192	4.43616	−1.257918
56	H	0.630145	4.086793	−1.907993
57	B	−0.23659	−0.016288	1.182006
58	Cl	−0.395661	0.020638	3.090256

Table Appx-4. Cartesian coordinates of the DFT optimized geometry of **6'*M*** at the B3LYP/6-311G(d,p).

	Symbol	X	Y	Z		Symbol	X	Y	Z
1	O	−4.006231	−1.506737	−0.57379	31	C	−3.06517	−5.564287	−0.634123
2	N	−1.175372	0.072354	0.736121	32	H	−2.853284	−6.62668	−0.645966
3	N	0.811272	−1.268946	0.599435	33	C	−2.085688	−4.670515	−0.230941
4	N	0.928854	1.118988	0.530282	34	H	−1.122273	−5.041103	0.094813
5	C	−1.83706	1.209223	0.34161	35	C	4.338638	−0.268074	−0.27847
6	C	−3.12829	0.786483	−0.146387	36	C	5.193027	−1.078828	0.484636
7	H	−3.889674	1.425894	−0.56072	37	H	4.796713	−1.595543	1.350922
8	C	−3.119284	−0.59858	−0.104433	38	C	6.540182	−1.200008	0.156815
9	C	−1.868403	−1.031013	0.403963	39	H	7.18592	−1.823482	0.764982
10	C	−1.319336	−2.303976	0.227308	40	C	7.059648	−0.517431	−0.941058
11	C	0.105252	−2.399061	0.249192	41	H	8.109127	−0.611673	−1.195713
12	C	1.067514	−3.300762	−0.306163	42	C	6.220249	0.283752	−1.712646
13	H	0.855206	−4.274832	−0.716626	43	H	6.612639	0.806481	−2.577835
14	C	2.292964	−2.660489	−0.333443	44	C	4.872739	0.40632	−1.387124
15	H	3.207351	−3.042815	−0.758866	45	H	4.220653	1.004195	−2.012385
16	C	2.116448	−1.341308	0.195769	46	C	−1.783152	3.673687	−0.136073
17	C	2.907619	−0.15548	0.089515	47	C	−2.850172	4.120481	0.658651
18	C	2.254815	1.095697	0.19031	48	H	−3.132785	3.547319	1.534056
19	C	2.572789	2.449761	−0.18687	49	C	−3.525047	5.29768	0.349466
20	H	3.54459	2.805086	−0.489687	50	H	−4.340698	5.630076	0.981771
21	C	1.409501	3.188263	−0.152788	51	C	−3.149864	6.050775	−0.761033
22	H	1.306346	4.226966	−0.422343	52	H	−3.674956	6.968267	−1.001095
23	C	0.331686	2.317273	0.248842	53	C	−2.098389	5.613906	−1.564326
24	C	−1.087335	2.412013	0.210326	54	H	−1.809893	6.185222	−2.439641
25	C	−2.319902	−3.284496	−0.191412	55	C	−1.423192	4.43616	−1.257918
26	C	−3.617515	−2.838359	−0.5613	56	H	−0.630145	4.086793	−1.907993
27	C	−4.600674	−3.73571	−0.967897	57	B	0.23659	−0.016288	1.182006
28	H	−5.570756	−3.342137	−1.244787	58	Cl	0.395661	0.020638	3.090256
29	C	−4.326188	−5.095102	−1.009314					
30	H	−5.097162	−5.788295	−1.323914					
